# CRISPR/Cas9 microinjection in oocytes disables pancreas development in sheep

**DOI:** 10.1038/s41598-017-17805-0

**Published:** 2017-12-12

**Authors:** Marcela Vilarino, Sheikh Tamir Rashid, Fabian Patrik Suchy, Bret Roberts McNabb, Talitha van der Meulen, Eli J. Fine, Syed Daniyal Ahsan, Nurlybek Mursaliyev, Vittorio Sebastiano, Santiago Sain Diab, Mark O. Huising, Hiromitsu Nakauchi, Pablo J. Ross

**Affiliations:** 1 Department of Animal Science, University of California Davis, Davis, CA United States; 2 Institute for Stem Cell Biology and Regenerative Medicine, School of Medicine, Stanford University, Stanford, CA United States; 3 Department of Population Health and Reproduction, School of Veterinary Medicine, University of California Davis, Davis, CA United States; 4 Department of Neurobiology, Physiology & Behavior, College of Biological Sciences, University of California Davis, Davis, CA United States; 5 Davis, California Animal Health and Food Safety Laboratory, University of California Davis, Davis, CA United States; 6 Centre for Stem Cells & Regenerative Medicine and Institute for Liver Studies, King’s College, London, UK

**Keywords:** Genetic engineering, Developmental biology

## Abstract

One of the ultimate goals of regenerative medicine is the generation of patient-specific organs from pluripotent stem cells (PSCs). Sheep are potential hosts for growing human organs through the technique of blastocyst complementation. We report here the creation of pancreatogenesis-disabled sheep by oocyte microinjection of CRISPR/Cas9 targeting *PDX1*, a critical gene for pancreas development. We compared the efficiency of target mutations after microinjecting the CRISPR/Cas9 system in metaphase II (MII) oocytes and zygote stage embryos. MII oocyte microinjection reduced lysis, improved blastocyst rate, increased the number of targeted bi-allelic mutations, and resulted in similar degree of mosaicism when compared to zygote microinjection. While the use of a single sgRNA was efficient at inducing mutated fetuses, the lack of complete gene inactivation resulted in animals with an intact pancreas. When using a dual sgRNA system, we achieved complete *PDX1* disruption. This PDX1^−/−^ fetus lacked a pancreas and provides the basis for the production of gene-edited sheep as a host for interspecies organ generation. In the future, combining gene editing with CRISPR/Cas9 and PSCs complementation could result in a powerful approach for human organ generation.

## Introduction

One of the main challenges of human organ transplantation is donor organ availability. *In vitro* creation of human-sized organs or tissues suitable for patient transplantation has proven difficult^1^. Interspecies blastocyst complementation provides an alternative approach and is based on emptying a “developmental organ niche” in one species by knocking out a specific gene, or genes, critical for development of a particular organ and using pluripotent stem cells (PSCs) from a different species to colonize the vacant niche and generate the desired organ^2^. As a proof of principle, the possibility for intra- and interspecies blastocyst complementation has been demonstrated using rodent models^3–5^. These results raised the possibility of generating functional human tissues and organs within an animal species (hosts) with similar anatomy, size, and physiology to humans^6^. Sheep fulfill these criteria, although some technical concerns such as efficient generation of gene knockout animals remain to be addressed.

*PDX1* (pancreatic and duodenal homeobox protein 1) is a Hox-type transcription factor that is involved in pancreas development in the mouse and rat^7^. Homozygous deficiency of *PDX1* in mice^3^, rats^5^, and pigs^8,9^ results in absence of pancreas development. However, the function of *PDX1* is uncharacterized in sheep. Recently, the rapid development of genetic engineering approaches such as the CRISPR/Cas9 (Clustered Regulatory Interspaced Short Palindromic Repeats – CRISPR associated protein) system produced an efficient system for editing specific genes in livestock animals. CRISPR/Cas9 is a protein-RNA complex with sequence-specific nuclease activity that generates a double strand break (DSB) at the target site in a very efficient manner. Binding specificity is achieved through base pairing of a single guide RNA (sgRNA) and target DNA sequence^10^. An error-prone non-homologous end-joining (NHEJ) mechanism often repairs the DSB DNA in the targeted region, leading to potential disruption of the protein coding sequence and inactivation of the gene. Using zygote microinjection and the CRISPR/Cas9 system, it is possible to produce gene-modified animals in a single step^11^. The high efficiency of CRISPR/Cas9 allows generation of bi-allelic mutants by direct zygote microinjection. However, in a high proportion of embryos, Cas9 exhibits a delayed activity and can result in mosaicism. It has been shown that CRISPR/Cas9 microinjection into zygotes can result in up to five mutant alleles in the same individual, which suggests that Cas9 is active during early embryonic cleavage stages^12^. A potential alternative to decrease mosaicism is to introduce CRISPR/Cas9 before DNA replication in the zygote, or even before fertilization^13^.

In this study, we evaluated if *PDX1* disruption can disable pancreas development in sheep. We studied the efficiency of MII microinjection as compared to zygote microinjection. Additionally, we used a dual sgRNA system and we achieved *PDX1* disruption as demonstrated by absence of pancreas development. Overall, we provide an effective and efficient approach for the production of gene-edited sheep that could be used for patient-specific human organ generation.

## Results

### CRISPR/Cas9 microinjection in MII oocytes results in improved outcomes when compared to zygote microinjection

**Figure 1:**
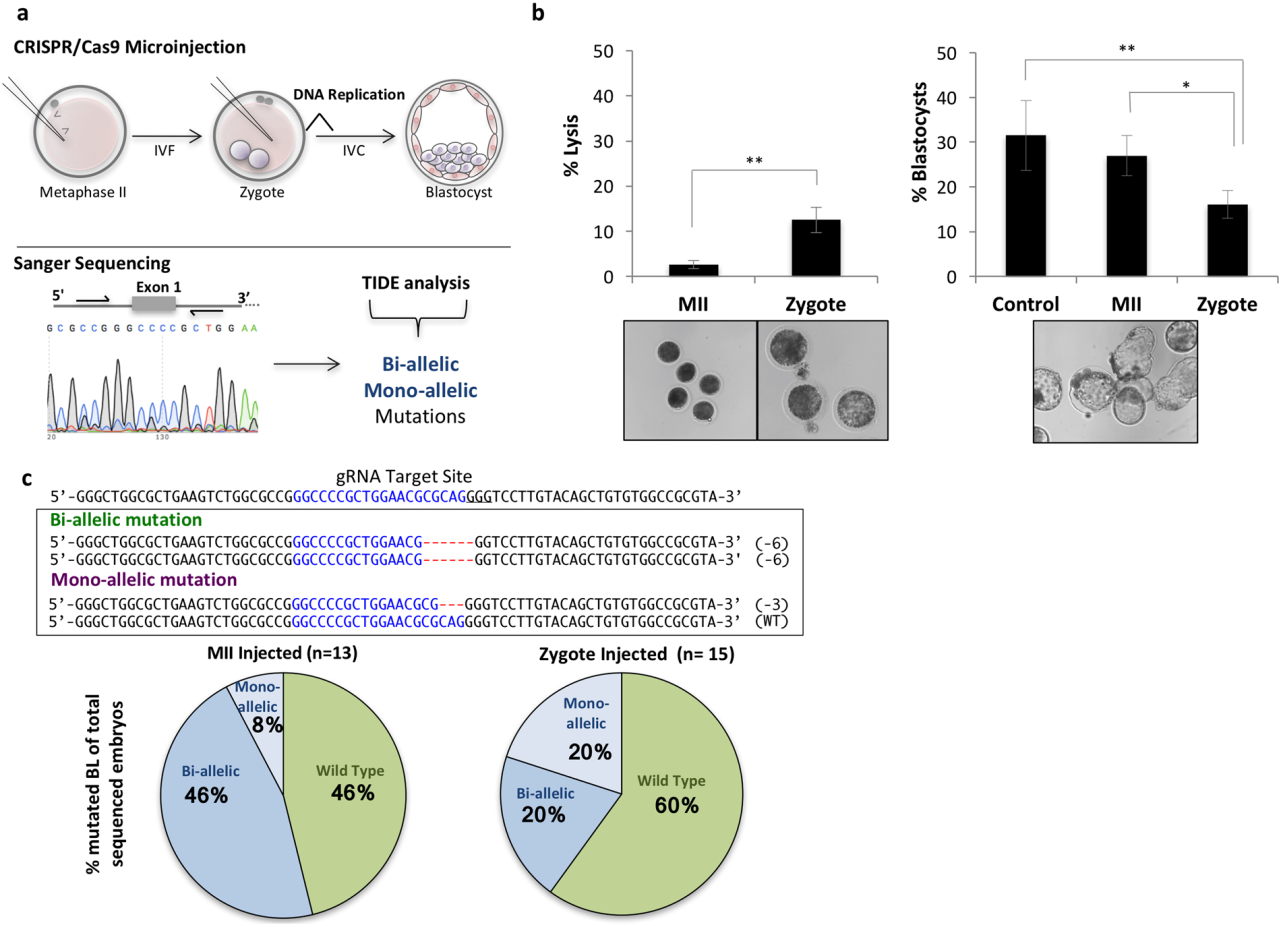
CRISPR/Cas9 microinjection of sheep oocytes and zygotes. (**a**) Schematic representation of CRISPR/Cas9 injection in sheep MII oocytes and zygotes. Presumptive embryos were *in vitro* cultured until the blastocyst stage for embryo genotyping by Sanger sequencing and mutations were determined using the TIDE bioinformatics package. (**b**) Lysis after microinjection of CRISPR/Cas9 was lower in MII oocytes than in zygotes. Development was higher after microinjection in MII oocytes and non-injected embryos compared to zygotes (*P < 0.05; **P < 0.05). (**c**) Sanger sequencing results from a bi-allelic and a mono-allelic mutant sheep blastocyst. The PAM sequence is underlined and the gRNA target region is shown in blue. Red dashes represent deletions. Mutation efficiency is presented in the pie chart. For MII injected oocytes they were 46% (6/13) bi-allelic mutated blastocyst and 20% (3/15) for zygote microinjected blastocysts.

We designed and produced a sgRNA targeting exon 1 of the *PDX1* gene. MII oocytes were denuded of cumulus oocyte complexes (COCs), microinjected with Cas9 mRNA and *PDX1* sgRNA, and parthenogenetically activated (PA) or *in vitro* fertilized (IVF). Presumptive zygotes were injected at 14 h post activation or insemination. Reduced lysis rates were observed in MII microinjected oocytes when compared to microinjected zygotes (2.6% (10/379) vs. 12.5% (47/376); p < 0.05). From surviving embryos, development to the blastocyst stage was higher in control (31.5%, 87/276) and MII-microinjected embryos (27.1%, 100/369) versus zygote-microinjected embryos (16.1%, 53/329; p < 0.05), irrespective of embryo production method (PA or IVF) (Fig. 1b; Supplementary information, Table S1).

**Figure 2:**
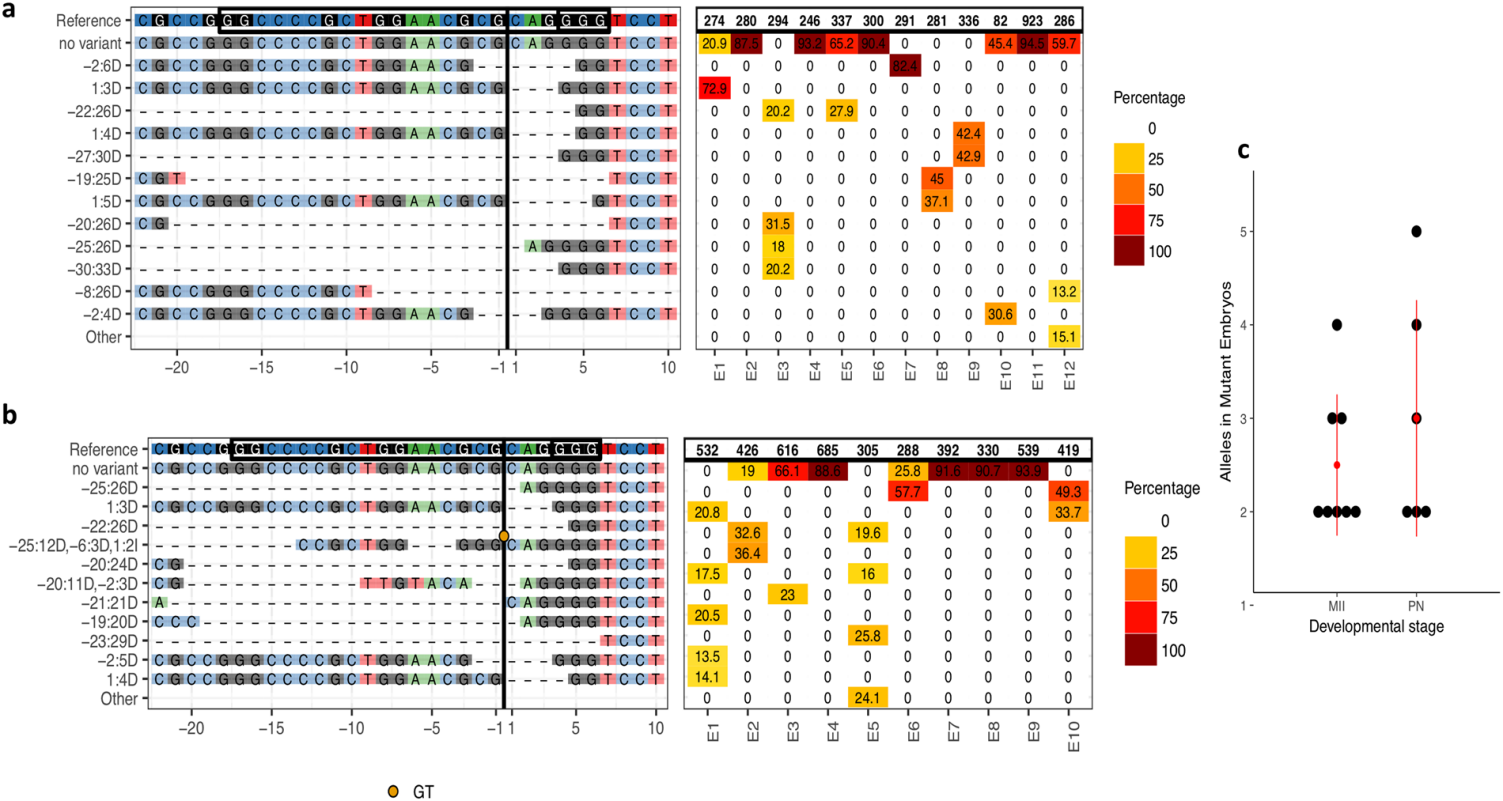
Deep sequencing reveals different mutations in sheep blastocysts microinjected with CRISPR/Cas9. Alignment of next-generation sequencing data obtained from sheep blastocysts injected with CRISPR/Cas9 targeting *PDX1* at MII oocytes (**a**) or at Zygotes (**b**). Mutations with frequencies higher than 12.5% were considered. Frequencies of alleles are shown in the HeatMap on the right panel and the top row refers to the total number of reads analyzed. Insertions with respect to the reference are presented underneath the alignments. **(c)** Number of alleles in mutant embryos after CRISPR/Cas9 microinjection in MII oocytes and Zygote.

Mutation efficiency was assessed by sequencing the target region in blastocyst stage embryos. The lack of wild type alleles was considered a bi-allelic mutation, where both maternal and paternal alleles are mutated even if they may harbor different types of mutations. An embryo was considered mosaic if three or more different alleles were present or when the ratio of one of the alleles was higher than 66%. Results showed that 53.8% of MII-injected (n = 13), and 40.0% of zygote-injected (n = 15) embryos had mutations, and 46.2% (6/13) and 20.0% (3/15) had bi-allelic mutations, respectively (Fig. 1c; Supplementary information, Fig. S1). To further study the nature of the targeted mutations, we performed deep sequencing of the CRISPR/Cas9 target site on blastocysts derived from MII (n = 12) and zygote (n = 10) microinjected oocytes and embryos. On average we analyzed 384 sequences per embryo (Fig. 2). We observed that 66.7% (8/12) and 60.0% (6/10) of the blastocysts microinjected at MII and zygote stages, respectively, had mutations. Blastocysts microinjected at MII stage had 25.0% bi-allelic mutations without mosaicism (3/12) as compared with 10.0% (1/10) when microinjected at the zygote stage. The presence of mosaicism among mutated alleles was not significantly different between groups (37.5%, 3/8; vs. 66.7%, 4/6 for MII and Zygotes, respectively). The average number of alleles per mutated embryo was 2.5 in MII microinjected oocytes compared to 3.0 in microinjected zygotes (not found to be statistically significant, p > 0.05). Additionally, we observed that most of the embryos with mutations had deletions whose lengths were a multiple of three, resulting in in-frame mutations possibly generating a functional PDX1 protein. As an example, oocyte E9 from MII microinjected group had a bi-allelic mutation, but allele #2 had a 30 bp in-frame deletion that would still allow the majority of the PDX1 protein sequence to be translated. Only embryo E8 from MII microinjected oocytes lacked any potentially functional allele, with one allele having a 25 bp deletion and the other a 5 bp deletion, both of which would result in disruption of the reading frame leading to expression of a truncated protein. Overall, CRISPR/Cas9 microinjection into sheep oocytes and embryos was efficient at inducing targeted mutations, with MII microinjections resulting in less lysis but higher developmental and bi-allelic mutation rates, while overall mutation efficiency and the degree of mosaicism was similar to zygote microinjection. Still, the high proportion of mutations that did not result in putative frame-shift mutations represents a concern for inactivating PDX1 using this approach.

### Targeting *PDX1* with a single sgRNA yielded in-frame mutations

**Figure 3:**
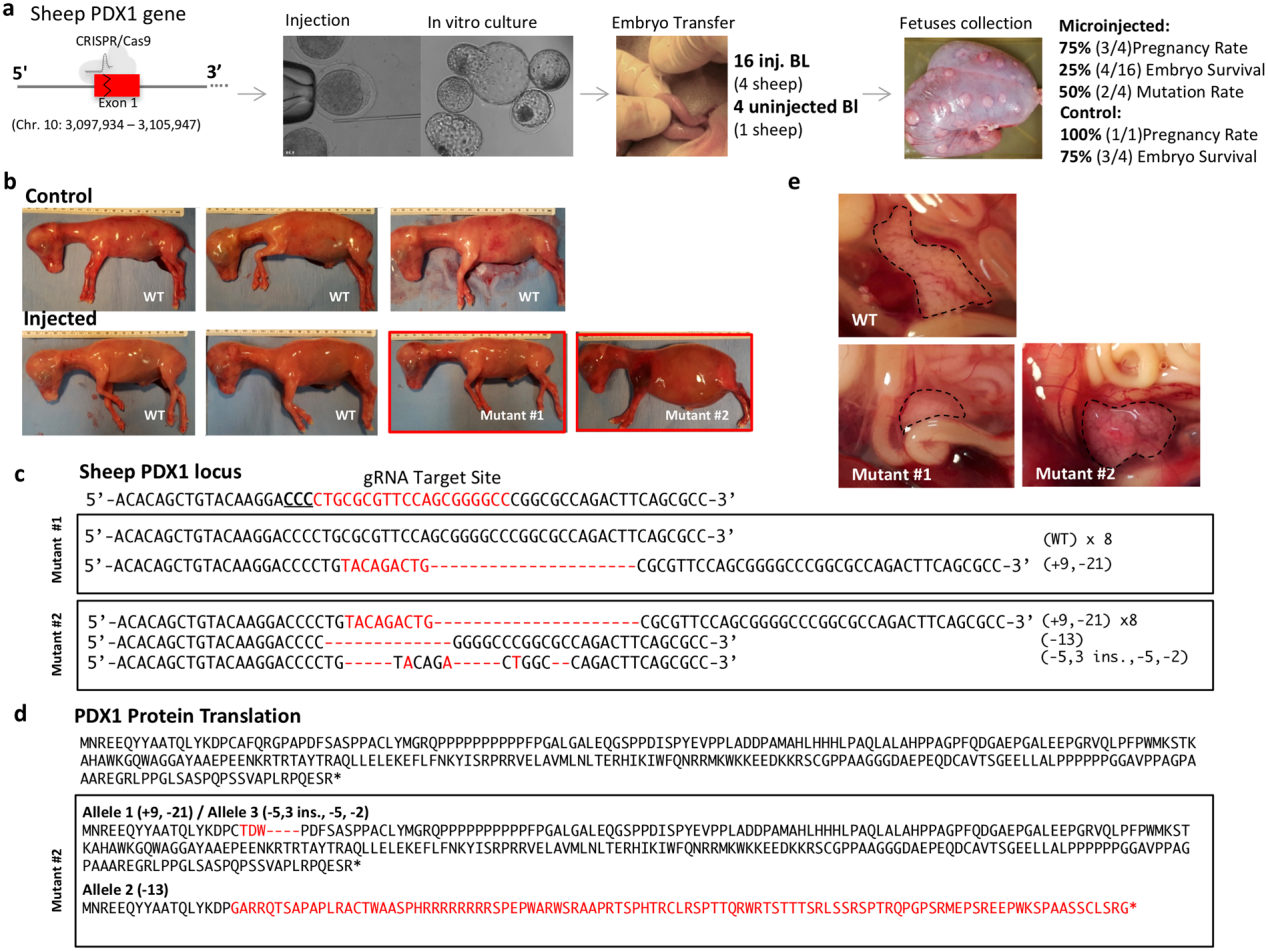
Targeting *PDX1* with a single sgRNA resulted in in-frame mutations. (**a**) Schematic representation of the sgRNA targeting *PDX1* gene in sheep. Sheep oocytes were microinjected with Cas9 mRNA and PDX1 sgRNA, cultured *in vitro*, and 16 injected blastocyst and 4 un-injected blastocysts were collected and transferred to 5 recipient ewes. (**b**) Seven fetuses (3 controls and 4 microinjected) were collected at day 75 of development and 2 of the microinjected fetuses had mutations. (**c**) Sequencing results from 9-10 colonies of each of the mutant fetuses after PCR and sub-cloning of the target region. Red dashes represent deletions and red letters insertions; insertions (+) or deletions (−) are shown to the right of each allele. The PAM sequence is underlined and the target region is shown in red. (**d**) Protein sequences of the Mutant#2 indicate that one of the mutant alleles only disrupted a few amino acids indicated in red, and therefore potentially active PDX1 might be present. (**e**) The pancreas was present in both mutant fetuses.

To test if *PDX1* knockout would alter pancreas development in sheep, we made embryo transfers of blastocysts that were injected with Cas9 mRNA and the *PDX1* sgRNA at the MII stage. Sixteen injected blastocysts were transferred to 4 recipient females and 4 non-injected blastocysts were transferred to a recipient female (control). At day 75, 3 of the ewes that received the microinjected blastocysts were pregnant, from which a total of 4 fetuses were recovered. The control ewe with non-injected blastocysts yielded 3 fetuses (Fig. 3a and b). Genomic DNA was extracted from the tail and liver of the collected fetuses, and the target region was amplified and sequenced. Two of the four CRISPR/Cas9 microinjected fetuses had mutations in the *PDX1* locus. TOPO-TA cloning was performed, and from the 9 clones sequenced in Mutant #1, only one had mutations while all 10 clones sequenced from Mutant #2 had mutations (Fig. 3c). Despite the lack of wild type alleles in Mutant #2, the mutated alleles did not generate a frame-shift. Consequently, the resultant mutant protein contained only a few different amino acids compared to the wild type protein and protein function was not disrupted (Fig. 3d). This result was confirmed by the presence of a pancreas in Mutant #2 (Fig. 3e). Furthermore, the size of the pancreas of the two mutant fetuses was within the range observed for controls (165 to 231 g). In summary, these results indicate that the use of our single sgRNA targeting *PDX1* is effective for inducing mutations, but not effective for generating functional knockouts given the potential high rate of in frame mutations.

### CRISPR/Cas9 microinjection using dual sgRNAs effectively knocks out *PDX1* and pancreatogenesis in sheep

**Figure 4:**
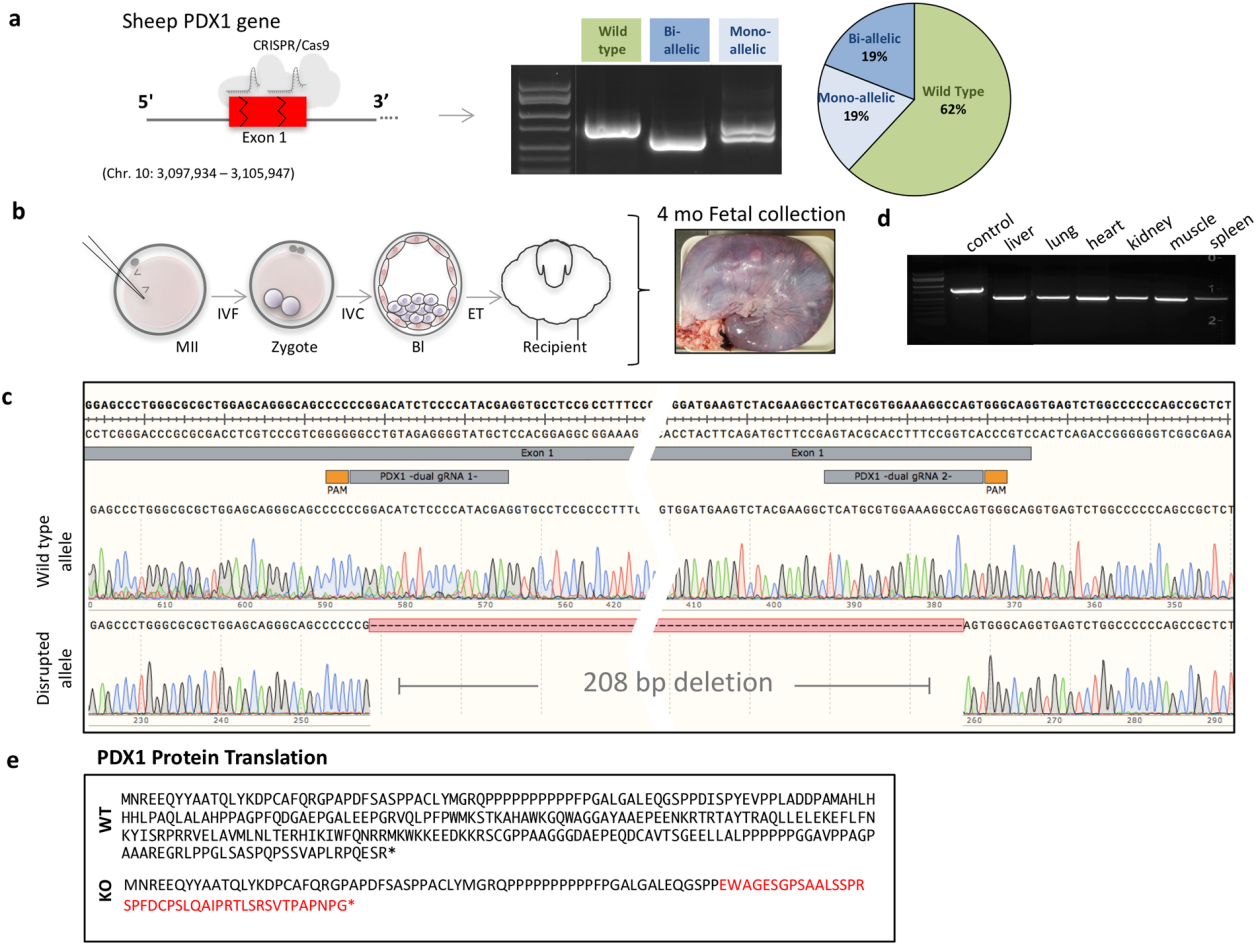
CRISPR/Cas9 using dual sgRNAs can effectively knockout *PDX1* in sheep. (**a**) Schematic representation of the two gRNAs designed to target *PDX1* loci in sheep. Dual sgRNA microinjection can induce a bi-allelic deletion that can be identified by PCR amplification and gel electrophoresis of the target region. The full-length gel is presented in the Supplementary Information. Mutation efficiency of the dual sgRNA microinjection is presented in the pie chart. From 21 microinjected oocytes 4 had mono-allelic and 4 had bi-allelic deletions. (**b**) Two sgRNAs targeting *PDX1* gene were microinjected into the MII II oocytes before IVF, cultured *in vitro* for 6 days and transfer to a recipient sheep. The fetus was collected at 4 months of gestation. (**c**) Genomic DNA was isolated and subjected to PCR, sub-cloning and Sanger sequencing. All of the sequenced colonies showed mutations with a 208 bp deletion. (**d**) Gel electrophoresis of PCR product -using specific primers for *PDX1-* from different tissues (liver, lung, heart, kidney, muscle and spleen) of the mutant fetus. Full-length gel is presented in the Supplementary information. **(e)** Protein sequence of the disrupted allele of the *PDX1*-KO sheep fetus is shown in red.

**Figure 5:**
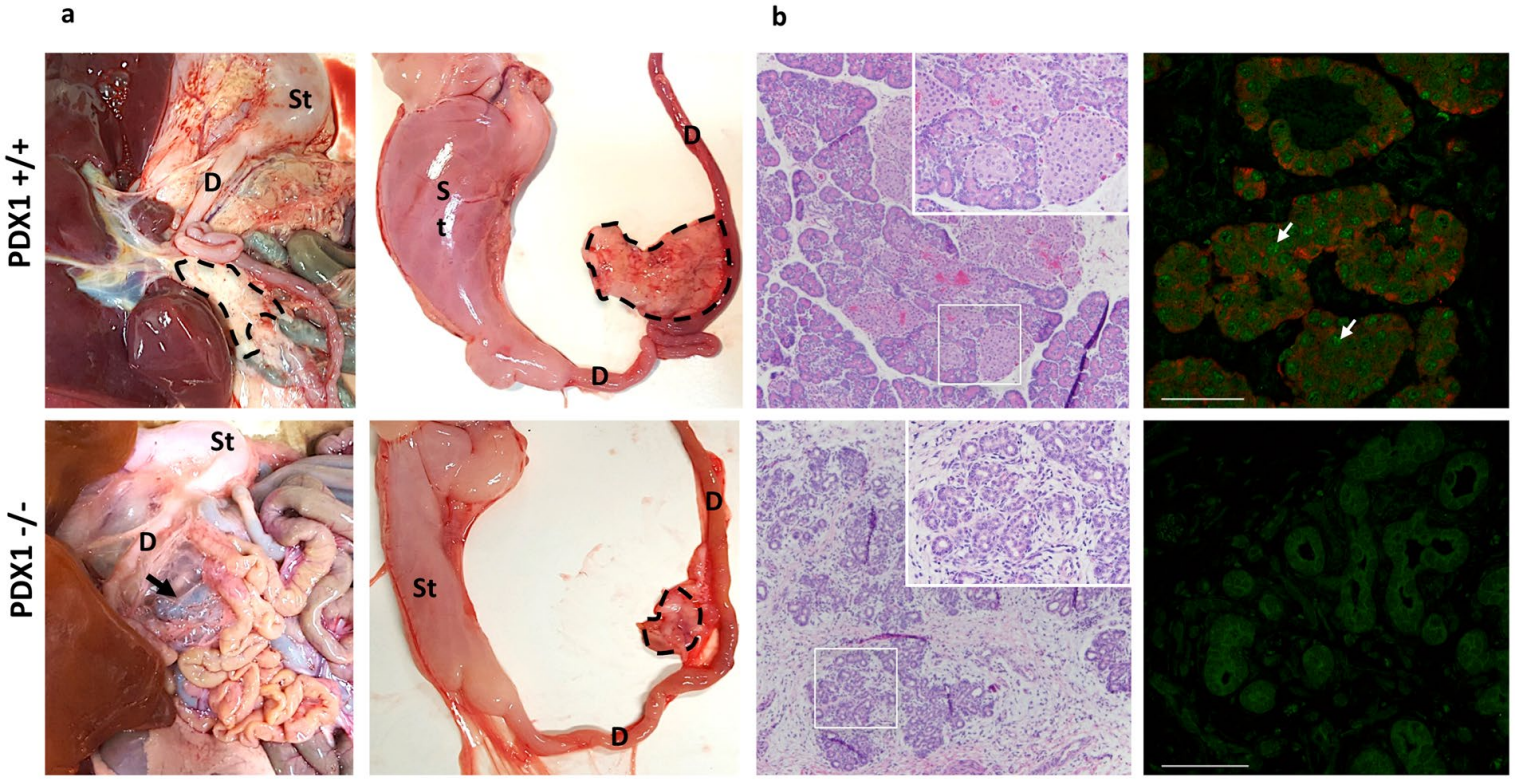
*PDX1*-KO phenotype in sheep. (**a**) Macroscopic appearance of the vestigial pancreas of a PDX1^−/−^ 4-month-old male fetus compared to a WT fetus of the same age. In the right panel the dashed lines indicate the pancreas (WT) and the vestigial pancreas (PDX1^−/−^) that were isolated for histology. St.: stomach; D.: duodenum. (**b**) Histology of the pancreas. The left panel shows representative images (40X) of the pancreas and vestigial structure stained with hematoxylin and eosin. Insets are high magnification images (200X) to illustrate the lack of Langerhans islets in the PDX1^−/−^ vestigial tissue. Right panel shows pancreatic tissue sections immunostained for PDX1 (green) and Insulin (red). The withe arrow indicates a *PDX1* positive cell. Bar indicates 50 µm.

Recently, induction of large gene deletions using CRISPR/Cas9 combined with two sgRNAs in pigs was demonstrated^9^. Based on these results we used a set of sgRNAs that when microinjected together are capable of inducing a 208 bp deletion in the coding region of *PDX1*; therefore increasing the probability of inactivating the PDX1 protein. Moreover, introducing a 208 bp deletion allows evaluation of mutation efficiency by gel electrophoresis of the PCR products without the need for sequencing (Fig. 4a). This approach was highly efficient for inducing mutations when injecting MII oocytes. From 21 microinjected oocytes, 19% (4/21) had mono-allelic and 19% (4/21) bi-allelic deletions (Fig. 4a). To test whether dual sgRNAs targeting *PDX1* can disrupt pancreas development, we transferred four microinjected embryos to a recipient ewe. At 4 months we collected the fetus (Fig. 4b) and genomic DNA was extracted. PCR and TOPO-TA cloning followed by sequencing of 10 colonies showed a large deletion in all sequenced clones (Fig. 4c). The large deletion was observed in genomic PCR from liver, lung, heart, kidney, muscle and spleen (Fig. 4d). This large deletion significantly altered the sequence of *PDX1*, resulting in deletion of 69 amino acids and a shift in the open reading frame resulting in a truncated product (Fig. 4e). Anatomic evaluation of the GI tract revealed the absence of the pancreas. Instead, a vestigial structure was present (Fig. 5a; Supplementary information, Fig. S2). Upon histological evaluation we observed the absence of islets of Langerhans (Fig. 5b). Immunofluorescence analysis confirmed the absence of PDX1 and insulin in *PDX1*-KO fetuses (Fig. 5b; Supplementary information, Fig. S3). These results demonstrate that, as in mice, rats and pigs, *PDX1* is necessary for pancreas development in sheep. CRISPR/Cas9 combined with dual sgRNAs is effective for gene disruption by direct oocyte microinjection.

## Discussion

Use of livestock species as hosts for human organ generation through blastocyst complementation is one of the main potential approaches that regenerative medicine could utilize. Since diabetes is a common disease that could be treated with stem cell-derived tissues, we chose to focus on pancreas development by targeting the *PDX1* gene. In agreement with previous studies, CRISPR/Cas9 was successfully used to generate target mutations in sheep^14–22^. We efficiently disrupted the *PDX1* gene in sheep by altering the protein coding sequence, resulting in an apancreatic phenotype.

Pigs and sheep are suitable models for human organ generation given their anatomical size, relatively short generation interval and easier handling as compared to other livestock animals. Recently, pancreatogenesis disabled pigs were produced by somatic cell nuclear transfer (SCNT) using transgenic fibroblasts as donor cells^8^ or using the CRISPR/Cas9 system^9^. However, until now there were no publications that describe this approach in sheep. The sheep also has proven to efficiently form an inter-species chimera with goats^23^.

Generation of mutant embryos by direct CRISPR/Cas9 microinjection into zygotes is typically performed prior to the onset of DNA replication to ensure only two copies of DNA are present. This is only possible in the short window between fertilization and DNA replication, which in cattle embryos lasts around 13 hours post-fertilization^24^. When gene editing occurs after DNA replication, it can lead to mosaicism. The presence of multiple alleles after using CRISPR/Cas9 was previously reported in sheep^14^ and other species^12,25^. Delayed activity of CRISPR/Cas9 is a likely cause for mosaicism. Therefore, we tested microinjecting CRISPR/Cas9 in MII oocytes, giving the system more time to edit targets prior to the onset of DNA replication. Even though the rate of mosaicism was not different between MII oocytes and zygotes microinjection, the proportion of bi-allelic mutations was higher after MII oocyte microinjection.

In the present study, we observed that cytoplasmic microinjection of MII oocytes reduces the lysis rate when compared to microinjection of zygotes. In livestock species visualization of the pronuclei is difficult because of the presence of lipid droplets that make the cytoplasm opaque^26^. This technical issue could explain the higher lysis rate when injecting zygotes, due to possible damage of the pronuclei when aspirating cytoplasm to break the plasma membrane. Our data also indicate that CRSPR/Cas9 MII oocyte microinjection increases blastocyst development rates versus zygote microinjection. This result is possibly also related to the technique of zygote microinjection in livestock species, and a reduction in damage of nuclear DNA in MII oocytes versus zygotes.

The ultimate goal of CRISPR/Cas9 microinjection is to induce gene mutations resulting in inactive gene products. Frame-shift mutations are an effective way to achieve gene inactivation via small deletions or insertions generally produced after CRISPR/Cas9 activity. Furthermore, bi-allelic mutations are required to assay the phenotype of knockout genes. We found that while microinjecting MII oocytes or zygotes induces similar overall mutation rates (~59%), MII microinjection quadrupled the presence of bi-allelic mutations (12% vs. 3%). Still, while using a single sgRNA resulted in high mutation efficiency, *PDX1* disruption is hard to predict due to in-frame mutations. In our study, generation of bi-allelic fetuses with in-frame mutations did not alter gene function as evidenced by normal pancreas development. The high frequency of in-frame mutations using the single sgRNA does not appear to arise from any obvious microhomologies around the mutation sites. As only one single sgRNA was tested extensively, it is unclear whether the high in-frame mutation rate is a property of all single sgRNAs in sheep (single sgRNAs in many other studies show no strong preference for in-frame mutations) or simply an aberrant attribute of the specific sgRNA we used. Using CRISPR/Cas9 with two sgRNAs has been shown to induce large gene deletions in other species^9,27,28^. As expected, the application of this strategy to target sheep *PDX1* induced a large *PDX1* deletion that resulted in the absence of pancreas development. Upon dissection, the area where the pancreas is located had a small structure with acinous-like tissue. Neither PDX1 nor insulin were detected in the tissue, confirming that islets were completely absent after *PDX1* disruption. Our results are supported by studies in mice where *PDX1* knockout resulted in a phenotype with the initial buds of the pancreas forming, but subsequent branching and morphogenesis arrested^29,30^. Additionally, the *PDX1*^+/−^ genotype did not appear to have an effect on pancreas development in sheep as has been previously shown in mice^31^.

While we did not test for off-target effects of the CRISPR/Cas9 approach, the fact that the same gene (*PDX1*) has been previously demonstrated in other species (mouse/rats/pig) to be essential for pancreatic development lends strong support to the notion that the CRISPR/Cas9-mediated mutations of the *PDX1* gene in the sheep embryos was the direct reason for the absence of the pancreas. Furthermore, given the intended use of this model – a host for interspecies organogenesis – off-target mutations that are not lethal/detrimental to the embryo would not represent a limitation for its use, as the product would be derived from the pluripotent cells and not the host animal.

In summary, we demonstrate that, as in other species, *PDX1* disruption in sheep leads to compromised pancreas development. The CRISPR/Cas9 system resulted in an efficient method for knockout generation in one step. Injecting MII oocytes reduced lysis after microinjection and improved development compared to zygote microinjection. In addition, bi-allelic mutation rates were improved. Finally, using a dual sgRNA injection strategy resulted in an efficient method for gene disruption in sheep. Overall, injecting MII oocytes with a dual sgRNA system improved development and mutation efficiency in sheep. In the future, combining gene editing by CRISPR/Cas9 with PSC injection could provide an interesting approach for human organ generation.

## Materials and Methods

### Animal care

All experiments involving animals were approved and performed in accordance with the University of California Davis Institutional Animal Care and Use Committee (IACUC Protocol #18343) and Stanford University IACUC (APLAC#29980). Recipient sheep were raised at the University of California, Davis Sheep Unit.

### General

All experiments were performed in accordance with relevant guidelines and regulations. All chemicals were purchased from Sigma-Aldrich Inc. unless otherwise specified.

### Sheep *in vitro* embryo production

Ovaries were collected from an abbatoir (Superior Farms, Dixon, California) and transported to the laboratory in saline solution at 37 °C. Oocyte aspiration was performed using a 21 G butterfly needle connected to a vacuum pump, aspirating from 2–6 mm antral follicles. Cumulus-oocyte-complexes (COC) were selected and *in vitro* maturation performed in groups of 30 COC in 60 µl drops of TCM199 supplemented with 10% Ovine Estrus Serum (OES), oFSH (50 ng/ml; National Hormone & Peptide Program, UCLA, CA), bLH (3 mg/ml; Sioux Biochemical), and cysteamine (0.1 mM). COC were matured for 24 h in 5% CO_2_ with humidified atmosphere at 38.5 °C. IVF was carried out using fresh semen immediately diluted with Andromed (Minitube) and selected by ascendant migration with a swim-up method using Fertilization Medium (SOF supplemented with 2% OES, 10 µg/mL heparin, 10 µg/mL hypotaurine). COCs were washed twice and placed in 60 µl drops of Fertilization media. Sperm concentration was adjusted to 1 × 10^6^ sperm/mL and oocytes were co-incubated with the sperm for ~14 hours in 5% CO_2_ with humidified atmosphere at 38.5 C°. Putative embryos were cultured in groups of 30 in 70 µL drops of KSOM (Evolve, Zenith Biotech) with 4 mg/mL of BSA under oil at 38.5 °C, 5% CO2 and 5% O2. Blastocysts were collected on Day 7 post-fertilization.

### Sheep parthenogenetic embryo production

Oocytes were collected and matured as described for *in vitro* embryo production. After maturation, the oocytes were denuded from the surrounding cumulus cells by vortexing in SOF-Hepes medium containing hyaluronidase (1 mg/mL) for 4 minutes. Denuded oocytes were washed with SOF-Hepes and exposed to 5 µM ionomycin (Calbiochem) in SOF-Hepes for four minutes. Oocytes were rinsed four times and incubated four hours in 2 mM of Dimethylaminopyridine (DMAP) in KSOM. After the incubation, oocytes were rinsed and cultured under the same conditions described above.

### sgRNA design and *in vitro* transcription

The single sgRNA targeting *PDX1* was designed and constructed by Transposagen (Kentucky, USA). The dual sgRNAs were designed using an online software (MIT CRISPR design tool) and synthetized using a cloning-free method. The oligos containing the sgRNAs and a T7 promoter were amplified by PCR using Q5 High-Fidelity DNA Polymerase (NEB) and purified (Macherey-Nagel). Guide RNA templates were used for *in vitro* transcription with the MEGAshortscript T7 Transcription Kit (Invitrogen) following the manufacturer’s instructions. sgRNA were purified using MEGAclearTM Kit Purification for Large Scale Transcription Reactions (Ambion) and dissolved in TE (Tris 10 mM, EDTA 0.1 mM) buffer for microinjection. The full nucleotide sequences of the oligos are provided in the supporting material (Supplementary information, Table S2). Cas9 mRNA was obtained from Sigma and diluted to 200 ng/µl using TE buffer.

### CRISPR/Cas9 microinjection

For zygote microinjection, presumptive embryos were used ~14 h post insemination/activation. For MII microinjection, oocytes were denuded of COCs by vortexing in SOF-Hepes with 1 mg/mL of hyaluronidase for 4 min. Microinjection was performed using an inverted microscope (Nikon, Tokyo, Japan) fitted with micromanipulators (Narishige, Tokyo, Japan) and two hydraulic oil microinjectors (Eppendorf, Hamburg, Germany). Cas9 mRNA (Sigma, 100 ng/µL) and sgRNA (50 ng/µL) were mixed and loaded to a 5–7 µm internal diameter blunt-end micropipette. Zygotes and MII oocytes were placed in 50 µL drops of SOF-Hepes supplemented with 10% of FBS, secured by a holding pipette and sgRNA and Cas9 mRNA were intra-cytoplasmically injected (5–10 pL) assisted by laser zona pellucida ablation (Saturn 5, RI, UK). The cytoplasm of the oocyte/zygote was aspirated by applying negative pressure to ensure membrane breakage^32^. After microinjection zygotes were returned to culture conditions and MII oocytes were *in vitro* fertilized or activated. All the microinjections were performed in groups of 25 and each session was limited to 30 min.

### DNA preparation and genotyping of a single blastocyst

Single blastocysts were lysed with 10 µl of lysis buffer (Epicentre) and incubated at 65 °C for 6 minutes and 98 °C for 2 minutes. Two rounds of PCR using GoTaq Hot Start Green Master Mix (Promega) with specific primers for *PDX1* sequences were performed. The PCR conditions were 95 °C for 5 min, followed by 35 cycles of 95 °C for 30 sec, 58 °C for 30 sec, 72 °C for 45 sec, followed by a final step of 72 °C for 10 min. PCR products were separated by gel electrophoresis, DNA bands were cut and purified using QIAquick Gel extraction kit (QIAGEN), and Sanger sequenced using the reverse primer (Quintara Biosciences). Sequences were aligned to the reference using SnapGene software. TIDE (Tracking of Indels by Decomposition) bioinformatics package^33^ was used to determine mutation efficiency. Primers for genotyping are provided in supporting material (Supplementary information, Table S2).

### Barcoded amplicon primer design, PCR amplification, and deep sequencing

Six different barcodes, each with a unique 16 bp sequence, were added to the forward and reverse primers for *PDX1* gene. DNA from single embryos was first PCR-amplified as described above, and a second round of PCR was performed using the barcoded primers. PCR products were checked for size (240 bp) using gel electrophoresis and all samples with the expected size were sent for library preparation and next generation sequencing using paired-end reads (2 × 150 bp) at the Center of Computational & Integrative Biology at Massachusetts General Hospital. De-multiplexing of barcodes was performed using a custom script. FastQ reads were mapped to the *Ovis aries* Pancreatic and duodenal homeobox 1 gene (GCA_000765115.1, NCBI) using BWA. Genomic variants were determined using the package CrisprVariant (Version 1.4.0)^34^.

### Sheep embryo transfer

Estrus synchronization was performed using a intravaginal progesterone device (0.3 g of progesterone; CIDR-G; Zoetis) for 6 days, followed injection of prostaglandin F2 (10 mg dinoprost thrometamine; Zoetis) and injection of PG600 (400 IU PMSG, 200 IU hCG; Intervet) coinciding with withdrawal of the device. Estrus detection was performed every 12 hours after CIDR-G withdrawal and embryo transfer was done 5 days after estrus. All the ewes were fasted for 16 hours before the procedure. Laparoscopic embryo transfer was performed in sedated ewes. Sedation consisted of the administration of 1.1–2.2 mg/kg of ketamine and 0.2-0.3 mg/kg of Midazolam 15 minutes before laparoscopy. Local anesthesia was done using 2% Lidocaine in the incision site. Embryos were transferred by laparoscopy (Karl Storz, Germany) to the tip of the uterine horn ipsilateral to the CL. Pregnancy was diagnosed 25 days after embryo transfer by transrectal ultrasonography (7.5 MgHz, Aloka 500).

### Fetus collection and genotyping

Recipient sheep were euthanized and fetuses collected at day 75 or 120 of gestation. Samples collected from different tissues were used for genomic DNA extraction using the DNeasy Blood & Tissue extraction kit (QIAGEN). PCR was performed using GoTaq Hot Start Green Master Mix (Promega) using the same primers and conditions described above. PCR products were purified, cloned into pCR^™^TOPO^®^TA vector (Life Technologies) and transformed into E. Coli DH5-alpha competent cells (NEB). Ten colonies were picked, cultured in LB broth, and plasmid DNA was extracted using a Miniprep kit (QIAGEN). Fast digest EcoRI (Thermo Scientific) was used to identify the positive colonies and samples were sent for Sanger sequencing (Quintarabio). Sequencing analysis was performed as described above.

### Histological analysis

Pancreas samples from the *PDX1* wild type and *PDX1*-KO fetuses were fixed with 4% paraformaldehyde at 4 °C overnight and then embedded in paraffin using standard procedures. Samples were cut into 7 µm slices and stained with Hematoxylin and Eosin. For immunofluorescence, samples were dewaxed, rehydrated and stained using specific antibodies. The primary antibodies used were rabbit anti-PDX1 (1:250, Abcam, ab47267) and guinea pig anti-Insulin (1:500, Dako, A0564).

### Statistical analysis

Lysis rate observed in microinjected oocytes and zygotes, *in vitro* embryo development on day 7 and the presence of mosaicism among experimental groups was analyzed by logistic regression including the effects of treatment and the replicate. The average number of alleles per mutated embryo was analyzed by one way ANOVA. Differences were considered significant when P < 0.05.

## Electronic supplementary material

Supplementary Information
