# Author Correction: CRISPR/Cas9 microinjection in oocytes disables pancreas development in sheep

**DOI:** 10.1038/s41598-020-64443-0

**Published:** 2020-05-05

**Authors:** Marcela Vilarino, Sheikh Tamir Rashid, Fabian Patrik Suchy, Bret Roberts McNabb, Talitha van der Meulen, Eli J. Fine, Syed Daniyal Ahsan, Nurlybek Mursaliyev, Vittorio Sebastiano, Santiago Sain Diab, Mark O. Huising, Hiromitsu Nakauchi, Pablo J. Ross

**Affiliations:** 1 Department of Animal Science, University of California Davis, Davis, CA United States; 2 Institute for Stem Cell Biology and Regenerative Medicine, School of Medicine, Stanford University, Stanford, CA United States; 3 Department of Population Health and Reproduction, School of Veterinary Medicine, University of California Davis, Davis, CA United States; 4 Department of Neurobiology, Physiology & Behavior, College of Biological Sciences, University of California Davis, Davis, CA United States; 5 Davis, California Animal Health and Food Safety Laboratory, University of California Davis, Davis, CA United States; 6 Centre for Stem Cells & Regenerative Medicine and Institute for Liver Studies, King’s College, London, UK

Correction to: *Scientific Reports* 10.1038/s41598-017-17805-0, published online 12 December 2017

The original version of this Article contained an error in the spelling of the author Syed Daniyal Ahsan, which was incorrectly given as Syed Ahsan. This has now been corrected in the PDF and HTML versions of the Article.

