# Supplementary material for: CRISPR/Cas9 microinjection in oocytes disables pancreas development in sheep
Source: Sci Rep. 2017 Dec 12;7:17472. doi: 10.1038/s41598-017-17805-0 (PMC5727233; doi:10.1038/s41598-017-17805-0)
Supplement: Supplementary file 1 — Supplementary Information [file 41598_2017_17805_MOESM1_ESM.pdf]

Supplementary Data

CRISPR/Cas9 microinjection in oocytes disables pancreas development in sheep

Marcela Vilarino[1,*], Sheikh Tamir Rashid[2,*], Fabian Patrik Suchy[2,*], Bret Roberts McNabb[3], Talitha van der Meulen[4], Eli J Fine[2], Syed Daniyal Ahsan[2], Nurlybek Mursaliyev[2], Vittorio Sebastiano[2], Santiago Sain Diab[5], Mark O. Huising[4], Hiromitsu Nakauchi[2, §], Pablo Juan Ross[1, §]

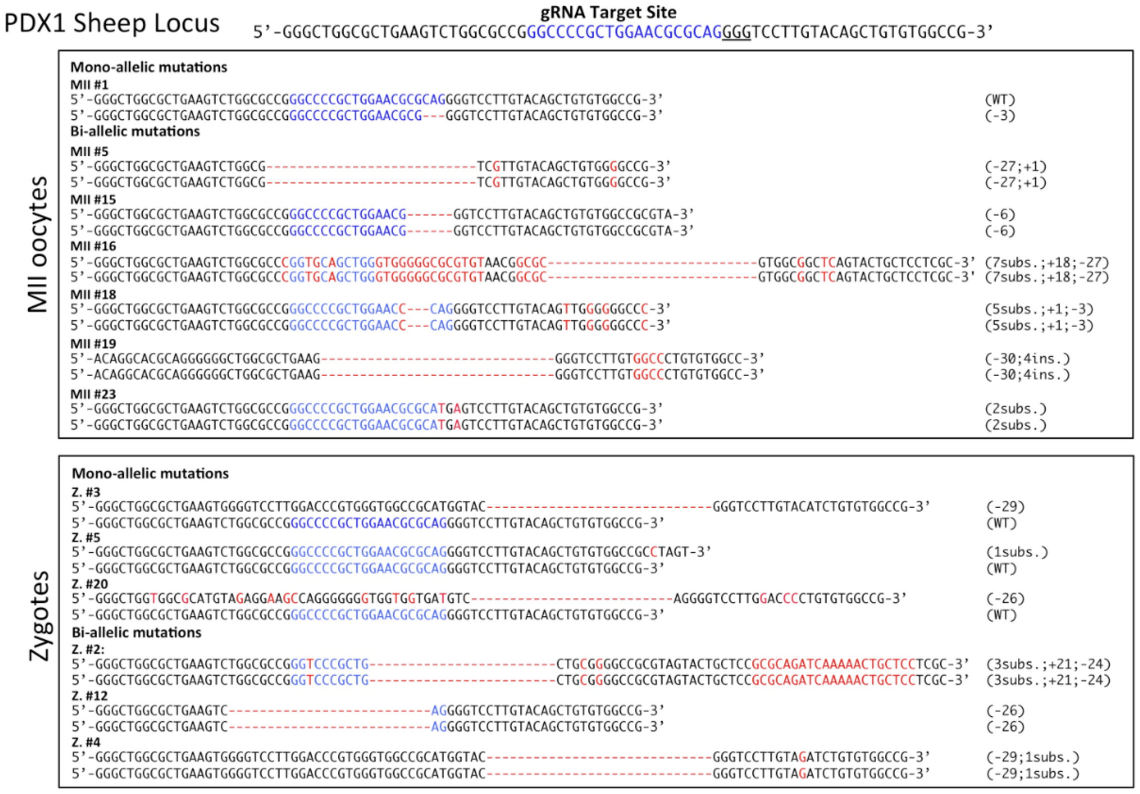

Supplementary Figure S1. Genotypes of the *PDX1* edited embryos injected at the MII oocyte or zygote stage. Sanger sequencing results from bi-allelic and a mono-allelic mutant sheep blastocyst are shown. The PAM sequence is underlined and the target region is shown in blue. Red dashes represent deletions and red letters insertions/substitutions.

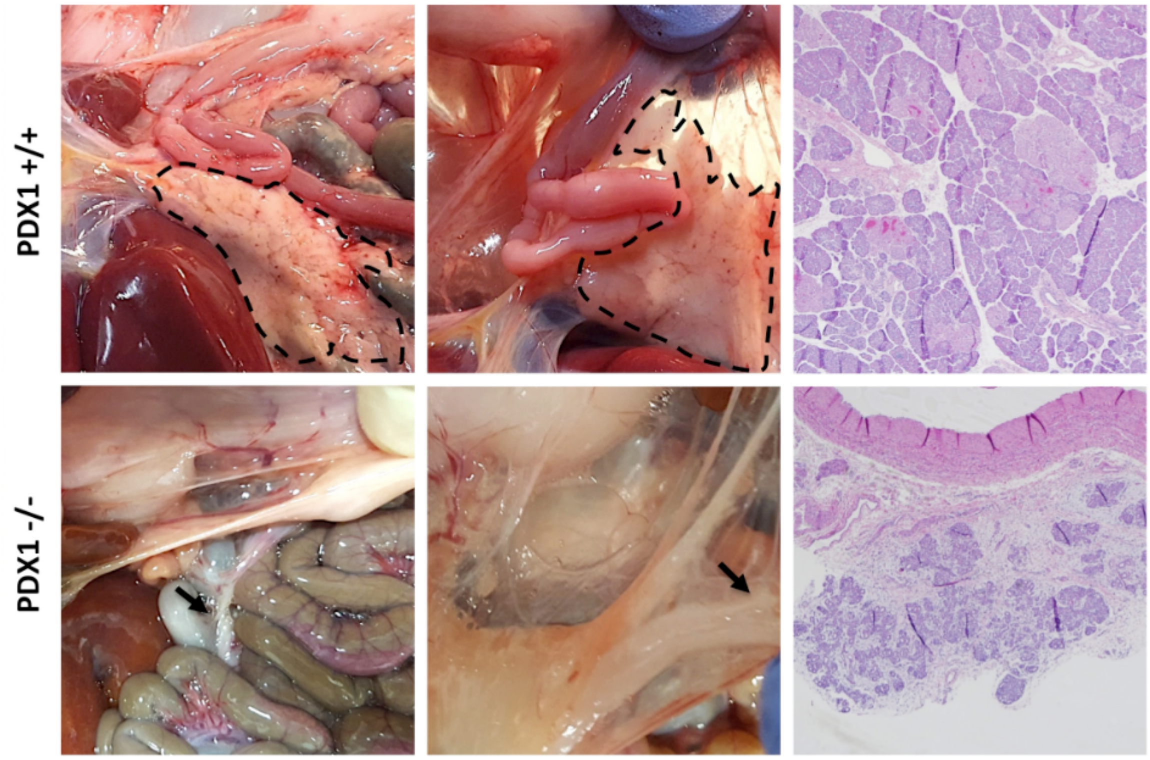

Supplementary Figure S2. The two left panels are macroscopic appearance of the vestigial pancreas (arrowheads) of the PDX1-KO fetus and the pancreas of a WT fetus (black dashes) at 4 months old of gestation. St.: stomach; D.: duodenum. The right panel is representative images (100X) of the pancreas and vestigial structure stained with hematoxylin and eosin.

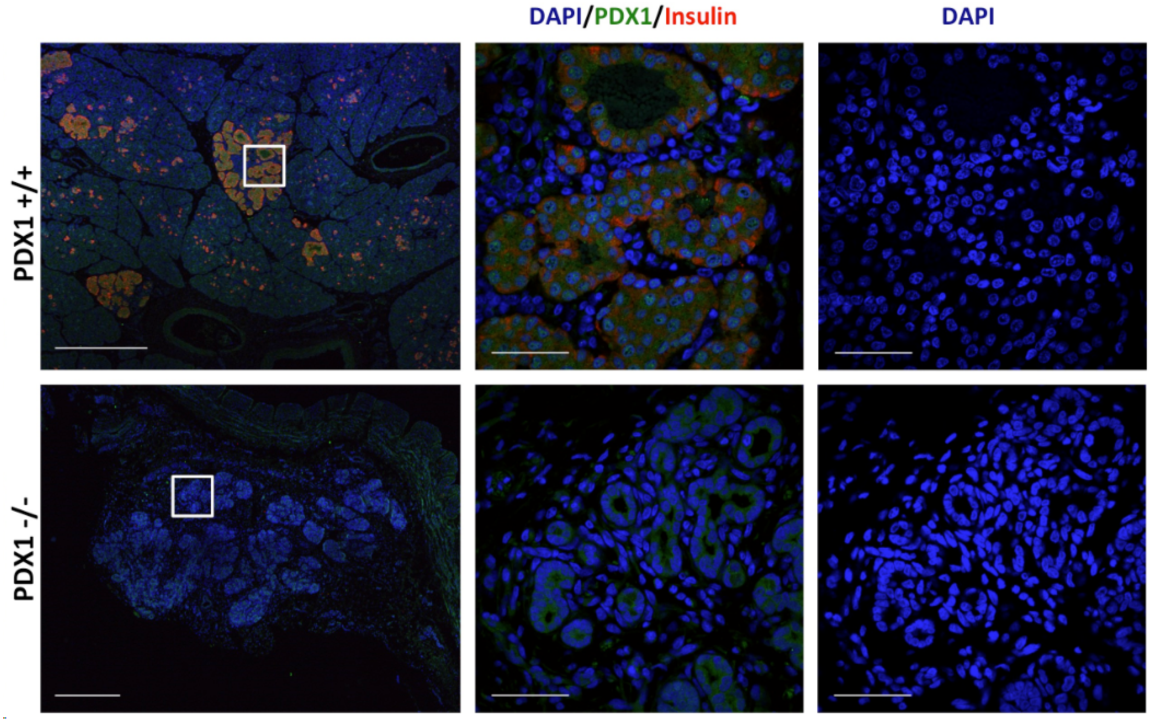

Supplementary Figure S3. Confocal microscopy of PDX1 (green) and Insulin (red) double immunostaining, and DAPI staining (blue) of a PDX1-KO 4 month-old fetus compared to a WT fetus of the same age. Scale bars in overviews 500 µm, in details 50 µm.

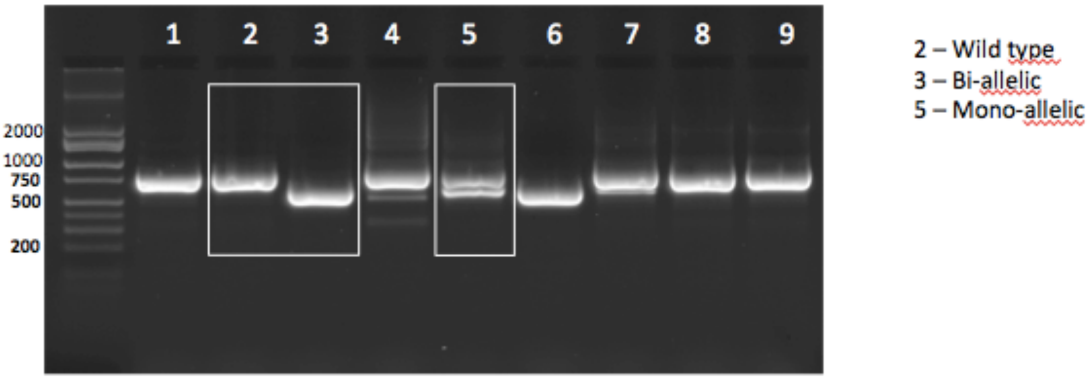

Full gel image for Figure 4a. Gel electrophoresis of PCR product of Sheep embryos injected with PDX1 sgRNA 1 & PDX1 sgRNA2.

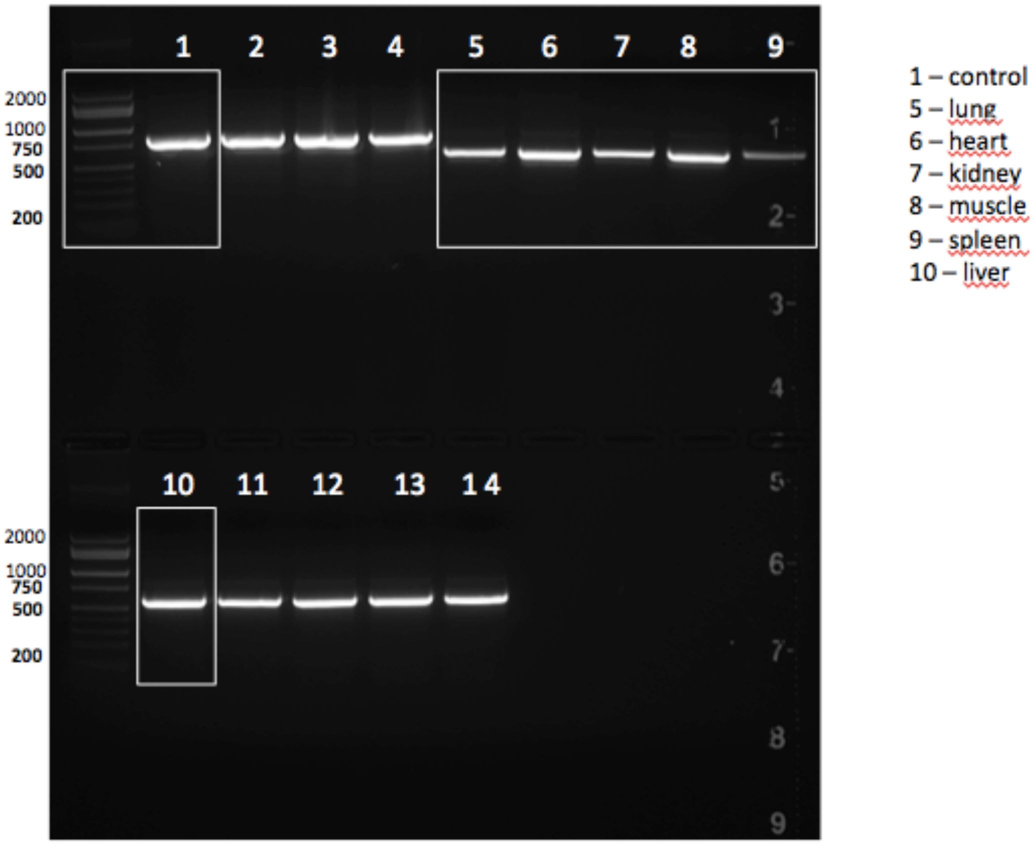

Full gel image for Figure 4d. Gel electrophoresis of PCR product -using specific primers for *PDX1*- from different tissues (liver, lung, heart, kidney, muscle and spleen) of the mutant fetus.

Table S1. Lysis and development rate after microinjection of MII oocytes and Zygotes. Embryos were produced by *in vitro* fertilization (IVF) or parthenogenetic activation (PA).

Replicate	IVP method	Group	Lysis rate (%)	Blastocyst rate (%)
1	IVF	Control		7/41 (17.1%)
		MII	4/82 (4.9%)	14/78 (17.9%)
		Zygote	13/74 (17.6%)	4/61 (6.6%)
2	IVF	Control		21/97 (21.6%)
		MII	3/66 (4.5%)	12/63 (19%)
		Zygote	7/67 (10.4%)	6/60 (10.0%)
3	IVF	Control		19/31 (61.3%)
		MII	1/58 (1.7%)	25/57 (43.9%)
		Zygote	14/85 (16.5%)	13/71 (18.3%)
4	PA	Control		20/50 (40%)
		MII	2/63 (3.2%)	20/61 (32.8%)
		Zygote	1/50 (2%)	9/49 (18.4%)
5	PA	Control		20/57 (35.1%)
		MII	0/110 (0%)	29/110 (26.4%)
		Zygote	12/100 (12%)	21/88 (23.9%)

Table S2. Oligos/Primers used in this study (5' – 3'). Underlined sequences are: gRNAs (Oligos for gRNA synthesis); and 16 bp barcodes (Primers for NGS).

**Oligos for gRNA synthesis**	
Oligo name	Sequence
oPDX1-single gRNA	GAAATTAATACGACTCACTATAGGGGGCCCCGCTGGAACGCGCAGGTTTTAGAGCTAGAAATAGC
oPDX1- dual gRNA1	TAATACGACTCACTATAGCGTACGGGGAGATGTCCGGGTTTTAGAGCTAGAAATAGC
oPDX1- dual gRNA2	TAATACGACTCACTATAGCACGCGTGGAAAGGCCAGTGTTTTAGAGCTAGAAATAGC
T7-Reverse constant	AAAAGCACCGACTCGGTGCCACTTTTTCAAGTTGATAACGGACTAGCCTTATTTTAACTTGCTATTTCTAGCTCTAAAAC
**Primers for PCR amplification**	
Primer name	Sequence
oPDX1-F	GAACCGCGAGGAGCAGTA
oPDX1-R-single gRNA	GAGCGGAGGCACCTCGTAT
oPDX1-R-dual gRNA	CGACGGCACTGAGGAGTC
**Primers for NGS**	
Primer name	Sequence
oPDX1-F-BC1	TCAGACGATGCGTCATGAACCGCGAGGAGCAGTA
oPDX1-F-BC17	CATAGCGACTATCGTGGAACCGCGAGGAGCAGTA
oPDX1-F-BC29	GCTCGACTGTGAGAGAGAACCGCGAGGAGCAGTA
oPDX1-F-BC34	ACTCTCGCTCTGTAGAGAACCGCGAGGAGCAGTA
oPDX1-F-BC38	TGCTCGCAGTATCACAGAACCGCGAGGAGCAGTA
oPDX1-F-BC40	CAGTGAGAGCGCGATAGAACCGCGAGGAGCAGTA
oPDX1-R-BC48	TCACACTCTAGAGCGAGAGCGGAGGCACCTCGTAT
oPDX1-R-BC52	GCAGACTCTCACACGCGAGCGGAGGCACCTCGTAT
oPDX1-R-BC54	GTGTGAGATATATATCGAGCGGAGGCACCTCGTAT
oPDX1-R-BC62	GACAGCATCTGCGCTCGAGCGGAGGCACCTCGTAT
oPDX1-R-BC70	CTGCGCAGTACGTGCAGAGCGGAGGCACCTCGTAT
oPDX1-R-BC09	CTGCGTGCTCTACGACGAGCGGAGGCACCTCGTAT
